# Alpha-Catulin Contributes to Drug-Resistance of Melanoma by Activating NF-κB and AP-1

**DOI:** 10.1371/journal.pone.0119402

**Published:** 2015-03-20

**Authors:** Birgit Kreiseder, Yvonne M Holper-Schichl, Barbara Muellauer, Nico Jacobi, Alexander Pretsch, Johannes A. Schmid, Rainer de Martin, Harald Hundsberger, Andreas Eger, Christoph Wiesner

**Affiliations:** 1 SeaLife Pharma GmbH, Tulln, Austria; 2 Department of Vascular Biology and Thrombosis Research, Center for Physiology and Pharmacology, Medical University of Vienna, Vienna, Austria; 3 Medical and Pharmaceutical Biotechnology, University of Applied Sciences, Krems, Austria; University of Queensland Diamantina Institute, AUSTRALIA

## Abstract

Melanoma is the most dangerous type of skin cancer accounting for 48,000 deaths worldwide each year and an average survival rate of about 6-10 months with conventional treatment. Tumor metastasis and chemoresistance of melanoma cells are reported as the main reasons for the insufficiency of currently available treatments for late stage melanoma. The cytoskeletal linker protein α-catulin (CTNNAL1) has been shown to be important in inflammation, apoptosis and cytoskeletal reorganization. Recently, we found an elevated expression of α-catulin in melanoma cells. Ectopic expression of α-catulin promoted melanoma progression and occurred concomitantly with the downregulation of E-cadherin and the upregulation of mesenchymal genes such as N-cadherin, Snail/Slug and the matrix metalloproteinases 2 and 9. In the current study we showed that α-catulin knockdown reduced NF-κB and AP-1 activity in malignant melanoma cells. Further, downregulation of α-catulin diminished ERK phosphorylation in malignant melanoma cells and sensitized them to treatment with chemotherapeutic drugs. In particular, cisplatin treatment led to decreased ERK-, JNK- and c-Jun phosphorylation in α-catulin knockdown melanoma cells, which was accompanied by enhanced apoptosis compared to control cells. Altogether, these results suggest that targeted inhibition of α-catulin may be used as a viable therapeutic strategy to chemosensitize melanoma cells to cisplatin by down-regulation of NF-κB and MAPK pathways.

## Introduction

Malignant melanoma is an aggressive and chemoresistant type of skin cancer that originates in melanocytes [1, 2]. Although less than 5% of skin cancers are melanoma, it causes a large majority of skin cancer related deaths [3]. The poor prognosis for late stage melanoma patients is due to the low response rates to conventional chemotherapy treatments with dacarbazine or its derivative temozolomide, that are below 20% [4, 5]. The platinum analog cisplatin is known to be highly effective against various solid tumors [6–8]. In malignant melanoma, the single-agent cisplatin reaches a response-rate of less than 10%. However, higher response rates have been reported in combinational therapy but without extending survival significantly due to greater toxicity [9]. Cisplatin mediates the activation of mitogen activated protein kinases (MAPK), nuclear factor kappa B (NF-ĸB), p53 and apoptotic pathways by interaction with DNA and forming DNA-adducts [6]. Induction of apoptosis is one of the main purposes of anti-cancer drugs, and therefore resistance to apoptosis is suggested as a possible mechanism leading to unresponsiveness of cancer cells [10, 11]. Enhanced NF-ĸΒ activity was found in many different cancers, including melanoma [12, 13]. NF-ĸΒ activation is induced by different stimuli including growth factors, cytokines, lymphokines, UV, stress, and pharmacological agents such as cisplatin [14]. The importance of NF-ĸB in tumorigenesis has been reasoned to its involvement in the regulation of inflammation, apoptosis, angiogenesis, tumor cell invasion and chemoresistance [15, 16]. Furthermore, there is ample evidence that MAPKs play a significant role in the activation of NF- ĸB [17], besides their normal role in the activation of the transcription factor activator protein-1 (AP-1) [18]. Concerning its oncogenic potential, AP-1 has been described as double edged sword because it may play a role in cell survival and apoptosis in cancer cells [19]. In this context, JNK and ERK have also been reported as both, pro- and anti-apoptotic regulators [20, 21].

α-Catulin is a cytoskeletal linker protein that stimulates the expression of anti-apoptotic genes [22, 23]. Previously, we demonstrated that α-catulin is an IKK-interacting protein that augments activation of NF-ĸB after stimulation with the inflammatory stimuli TNF-α or IL-1 as well as the activation of the Rho signaling pathway in HeLa and HEK293 cells [23]. Recently, we showed that α-catulin is highly expressed in malignant melanoma cells compared to melanocytes and that α-catulin is a key driver of tumor progression, invasion and metastasis via the upregulation of E-cadherin and the downregulation of mesenchymal genes such as N-cadherin, Snail/Slug and the matrix metalloproteinases 2 and 9 [24]. Accordingly, Cao et al. (2012) reported that α-catulin was highly expressed in squamous cell carcinoma and its knockdown decreased the migratory and invasive behavior in both tumor cells *in vitro* and in xenotransplants *in vivo* [25]. In addition, it has been shown that α-catulin increased NF-ĸB through an ILK-dependent pathway leading to elevated fibronectin and integrin α_v_β_3_ expression and therefore promoted tumor cell migration, invasion and metastasis in lung cancer cells [26].

In this study we show for the first time, that down-regulation of α-catulin diminished NF-ĸB, MAPK and AP-1 activation in malignant melanoma cells. Furthermore, α-catulin knockdown sensitized melanoma cells to treatment with cisplatin and other chemotherapeutic drugs. Cisplatin treatment decreased ERK-, JNK- and c-Jun activity in α-catulin knockdown melanoma cells, which was accompanied by reduced cell proliferation and enhanced apoptosis compared to control cells.

## Materials and Methods

### Biochemicals and Antibodies

Purified non-labeled mouse/rabbit mono-/polyclonal antibodies were anti-CTNNAL1, Mcl-1, CBP, p38 and p-p38 (Abcam, Cambridge, MA, USA), ERK, p-ERK, JNK, p-JNK, c-Jun, p-cJun, p21^waf/cip1^, p53, GAPDH, (Cell Signaling Technology, Inc., Danvers, MA, USA), KI-67 (Santa Cruz, Texas, USA). HRP-conjugated secondary anti-mouse and anti-rabbit antibodies were obtained from Life Technologies and Cell Signaling Technology, Inc., respectively. Anti-α-tubulin-HRP conjugated antibody was purchased from Cell Signaling Technology, Inc.

Texas-Red conjugated secondary anti-mouse and anti-rabbit antibodies were purchased by Jackson Immuno (Newmarket, UK). HGF and TNF-α were purchased from PeproTech Inc. (Rocky Hill, NJ). LPS was purchased from Sigma-Aldrich (St. Louis, MO, USA).

5x NFκB-luc-Reporter and AP-1-luc-Reporter plasmids were obtained from Agilent technologies (Santa Clara, USA), pGL3 reporter and control vectors were purchased from Invitrogen, E-cadherin si-RNA was purchased from Santa Cruz Biotechnology, Inc. and Caspase3/7, 8 and 9 assay and CellTiter-Blue cell viability Assay from Promega (Madison, USA). Staurosporine was purchased from eBioscience (CA, USA). Chemotherapeutic agents dacarbazine, cisplatin and paclitaxel were acquired from Sigma-Aldrich.

### Cells and cell cultures

Human metastatic melanoma cells from spleen (Mel.7), skin (Mel.17) and lymph node (Mel.15) were isolated and cultivated as described previously [27]. Human melanoma cells A375 were purchased from Sigma-Aldrich and cultivated in DMEM (Invitrogen). Human primary melanocytes were obtained from Provitro and cultivated in melanocyte growth medium (Provitro, Germany) with 10% FCS (PAA Laboratories, Pasching, Austria) and 1% Penicillin/Streptomycin (Sigma-Aldrich). In all experiments cell culture medium was supplemented with 10% FCS and 1% Pen/Strep unless indicated otherwise in the figure description.

Spheroids were generated by hanging drop method with 100 cells per drop and after four days of incubation spheroids were used for viability assay and microscopy.

### Quantitative Real-time PCR

Total RNA was extracted using the RNeasy Mini Kit (Qiagen, USA). RNA was reverse-transcribed with the First strand cDNA synthesis Kit (Roche Diagnostics, Germany) according to manufacturer’s instruction. Real Time PCR was performed with TaqMan Gene expression Master Mix, Assay, Primer and Probes (HS00972094 CTNNAL1, HS99999905 GAPDH; Applied Biosystems, USA). Reactions were run on the Light Cycler 480, Roche. Threshold cycle (Ct) values of the target genes were converted to arbitrary expression values by extrapolation from the standard curve and finally normalized to the internal control.

### Western blotting

For Western blotting, proteins were extracted from 10^5^ cells from each cell line. Total protein extracts were separated by 4–20% SDS-PAGE (Peqlab, Erlangen, Germany) and transferred with Trans-Blot Turbo, Transfer System to a Trans-Blot Turbo TransferPack Mini Format, 0,2 μm PVDF membrane (BioRad, California, USA). Membranes were blocked with 5% nonfat milk in Tris-buffered saline pH7,4 (TBS, BioRad), and immunodetection was carried out using specific antibodies (see Biochemicals and Antibodies) via chemiluminescence with ChemiDoc MP Imaging System, Universal Hood III (BioRad).

### Expression vectors and lentiviral infection

Lentiviral vector constructs pLEX-MCS, pLEX-JRED and pGIPZ shRNAmir CTNNAL1 (Table 1) and pGIPZ nonsilencing control were purchased from Thermo Scientific. Myc-catulin vector was described previously [24]. For stable transfection, 5 x 10^6^ HEK293T cells were used for the production of lentiviral stocks with a translentiviral packaging system (Thermo scientific). Melanoma cells (2 x 10^5^) were infected with lentiviral stocks and selected with puromycin [24]. Sequences of shRNAmir CTNNAL1 RNAs (Open Biosystems) were as follows: V3LHS_356693 here called sh-catu1, sense strand 5´-AGCTCAAAGCAAGAAAACA-3´; antisense 5´-GTTTTCTTGCTTTGAGCT-3; sh-catu2 (V3LHS_356695), sense strand 5´-AGCTTGTTGAGACCTGTCG-3; antisense: 5´-CGACAGGTCTCAACAAGCT-3´.

### Cell transfection

4x10^5^ melanocytes or melanoma cells per well were transiently transfected by the lipofectamin-2000 method using 2μg DNA and 7μl lipofectamin (Life Technologies). After 6 hours transfected cells were rinsed and incubated for additional 16–48 hours before they were used for the experiments.

### Luciferase Reporter gene assay

A 5x NF-κB-Luc reporter gene was transfected into melanocytes or stable transfected melanoma cells (sh-catu2 and n.s.). Cells were either co-transfected with IKK-β or p65 and/or stimulated with TNF-α, LPS, HGF or 10% FCS. Luciferase levels were normalized for a co-transfected JRED control and luciferase values were determined and normalized for co-transfected RFP values.

An AP-1-luc reporter gene was transfected into stable infected melanoma cells (sh-catu2 and n.s.) Cells were non-stimulated or stimulated with TNF-α or LPS luciferase levels were detected using Infinite F200 PRO multiplate reader (TECAN, Männedorf, Switzerland) and normalized for a co-transfected JRED control.

### Flow Cytometry analysis

For cell cycle distribution analysis Melanoma 7 cells (n.s., sh-catu2) were treated with cisplatin for 48 hours, detached, washed with PBS and fixed with 70% ice cold Ethanol. Then cells were washed again with PBS and stained with propidium-iodide solution (0.1% Triton-X-100, 2 mg DNAse free RNAse A and 500 μg/ml propidium-iodide) for 15 min at 37°C. Thereafter flow cytometry analysis was performed using Accuri Flow Cytometer and CFlow plus software (BD Biosciences).

For apoptosis-necrosis detection Melanoma 7 cells (n.s., sh-caut1 and 2) were treated with cisplatin for 48 h. Then cells were detached, centrifuged and washed with ice cold PBS. Thereafter cells were stained with APC-Annexin V (BD-Pharmingten) in 1x binding buffer (BD-Pharmingten) for 30 min at 4°C. Then Propidium Iodide Solution (Sigma-Aldrich) was added and Flow cytometry analysis was performed using Accuri Flow Cytometer and CFlow plus software (BD Biosciences).

Cytochrome c release assay was performed with the InnoCyte Flow Cytometric cytochrome c release Kit from Millipore (Billerica, MA, USA). Melanoma 7 cells (sh-catu2 and n.s.) were treated with different concentrations of cisplatin for 6 hours. Permeabilization, fixation and staining of the cells were performed according to the manufacturer’s instructions. Cells were analyzed using Accuri Flow Cytometer and CFlow plus software (BD Biosciences).

### Cell viability assays and apoptosis

Stable infected Mel.7, Mel.17 and Mel.15 cells (n.s., sh-catu2) were treated with different concentrations of cisplatin, dacarbazine, paclitaxel or staurosporin for 48h, cell survival normalized to untreated cells (pos. contr.) and analyzed by CellTiter-Blue cell viability assay substrate. After 2 hours of incubation the cells were analyzed with Infinite F200 PRO multiplate reader (TECAN).

Caspase-Glo 3/7, 8 and 9 Assay from Promega was used to determine cells in apoptosis. Cells were treated as in the cell viability assay and luciferase substrate was added after 24 h. Luciferase was detected with Infinite F200 PRO multiplate reader (TECAN) and normalized for GFP values (stable infected).

JC-1—Mitochondrial Membrane Potential Assay Kit (Abcam) was used to investigate the level of cells in apoptosis. Stable infected Mel.7 cells (n.s., sh-catu2) were treated with different concentrations of cisplatin for 6 h and stained with JC-1 solution for 10 min. Relative fluorescence level was detected at a wavelength of 535 nm with Infinite F200 PRO multiplate reader (TECAN) and normalized for GFP values (488 nm).

### Cell proliferation assay

Stable infected Mel.7 cells (n.s., sh-catu2) were seeded in 96 well plates at an amount of 2500 cells per well. After 24 hours cells were treated with 20, 10, or 5 μg/ml cisplatin or were left untreated and after 48 hours CytoSelect BrdU Cell Proliferation ELISA Kit (Cell Biolabs, Inc.) was used to determine cell proliferation. Therefore, cells were treated with BrdU solution, fixed and stained with antibodies against BrdU (provided in the kit) and detected with the multiplate reader at 450 nm wavelength. BrdU values were normalized to gfp values measured before treatment with BrdU solution.

### Statistical analysis

The Student's paired *t*-test was used. Reported *P* values are three-tailed and *P* < 0.05 (*) was considered statistically significant, P<0.01 (**) and P<0.001 (***) was considered as statistically highly significant.

## Results

### α-Catulin increases NF-κB activation in malignant melanoma cells

**Fig 1:**
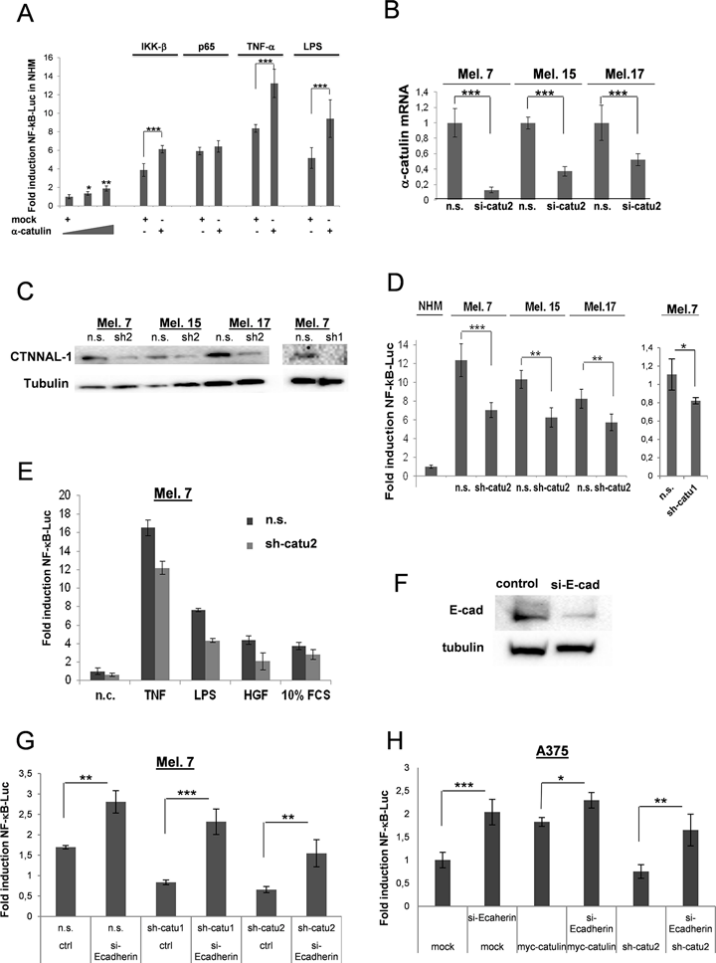
α-Catulin promotes NF-κB activation in human primary melanocytes and melanoma cells. (**A**) A 5x NF-B-luc reporter gene (0.25μg) was co-transfected into melanocytes together with different concentrations of α-catulin (1 or 1.5 μg) with or without IKK-β(0.5μg) or p65 (0.5μg). 24 h later cells were non-stimulated or stimulated with TNF-α or LPS. Luciferase levels were normalized for a co-transfected RFP control (0.25μg). (**B**) Mel.7, Mel.15 and Mel.17 cells were stable infected with lentiviral particles containing a vector-based mirRNA construct directed against α-catulin (sh-catu 1 or sh-catu2; Materials and Methods), and α-catulin mRNA levels were analyzed by real-time PCR. (**C**) Mel.7, Mel.15 and Mel.17 cells were analyzed by Western blot with antibodies against α-catulin. (**D**) A NF-κB-luc reporter gene was tranfected into melanocytes and different melanoma cells containing stable integrated α-catulin mirRNA constructs (α-catulin-knockdown), and luciferase values were determined 24 h later and normalized for co-transfected JRED values. (**E**) Melanoma 7 cells as in (**D**) except that the cells were stimulated with TNFα, LPS, HGF and 10% FCS for further 8 h. (**F**) Mel.7 cells (n.s., sh-catu1/2) were transfected with NF-κB reporter plasmid and with or without si-RNA construct directed against E-cadherin and luciferase values were determined. (**G**) NF-κB-luc reporter assay with A375 melanoma cells transfected with mock, myc-α-catulin or sh-catu2 plasmids together with or without E-cadherin si-RNA. *Indicates P>0.005, **P>0.001, ***P>0.0001, Student´s *t* test.

NF-ĸB activity has been shown to be upregulated in melanoma cells [17]. Previously, we demonstrated that α-catulin is an IKK-interacting protein that augments activation of NF-ĸB after stimulation with the inflammatory stimuli TNF-α or IL-1 as well as the activation of the Rho signaling pathway in HeLa and HEK293 cells [23]. To examine whether α-catulin can enhance NF-ĸB activity in melanocytes, we transfected normal human melanocytes (NHM) cells with a NF-ĸB reporter gene (5x NF-ĸB-Luc) plus different concentrations of α-catulin or mock, and either together with IKKβ or p65 expression vectors (S1 Fig.), or stimulated the cells with TNFα or LPS. We found that α-catulin increased already the basal NF-ĸB activity in melanocytes in a concentration dependent manner, but further augmented NF-ĸB activation in a highly significant manner when cotransfected with IKKβ or stimulated with TNFα or LPS for 8 h (Fig. 1A). In contrast, p65-mediated activity of the reporter was not enhanced by α-catulin. To further substantiate our findings, we knocked down the expression of α-catulin using lentiviral vectors in three different freshly isolated melanoma cells (Fig. 1B and C). Down-regulation of α-catulin significantly decreased the level of the NF-ĸB-luciferase reporter in all three melanoma cell lines (Fig. 1D; n.s. versus sh-catu1 and sh-catu2). α-Catulin knockdown also decreased NF-ĸB activity after TNFα-, LPS-, HGF- and Serum (10% FCS) activation in Melanoma 7 cells (Fig. 1E), which is consistent with our previous finding that α-catulin is central for mediating NF-ĸB activation. Previously, it was demonstrated that α-catulin regulates E-cadherin and that E-cadherin regulates NF-κB and AP-1/c-JUN [24, 53, 54]. To examine whether α-catulin and E-cadherin have synergistic effects melanoma cells were transfected with a NF-ĸB reporter gene (5x NF-ĸB-Luc) plus sh-catu (downregulation) or myc-α-catulin (ectopic expression) together with or without si-E-cadherin RNA (Fig. 1F). Interestingly, E-cadherin silencing significantly increased the low (sh-catu-mediated) NF-ĸB-luciferase reporter level whereas the elevated NF-κB activity caused by myc-α-catulin was further enhanced after E-cadherin silencing (Fig. 1G and H), suggesting that α-catulin increases NF-κB expression via downregulation of E-cadherin.

### Down-regulation of α-catulin diminishes AP-1 activity and ERK phosphorylation in malignant melanoma cells

**Fig 2:**
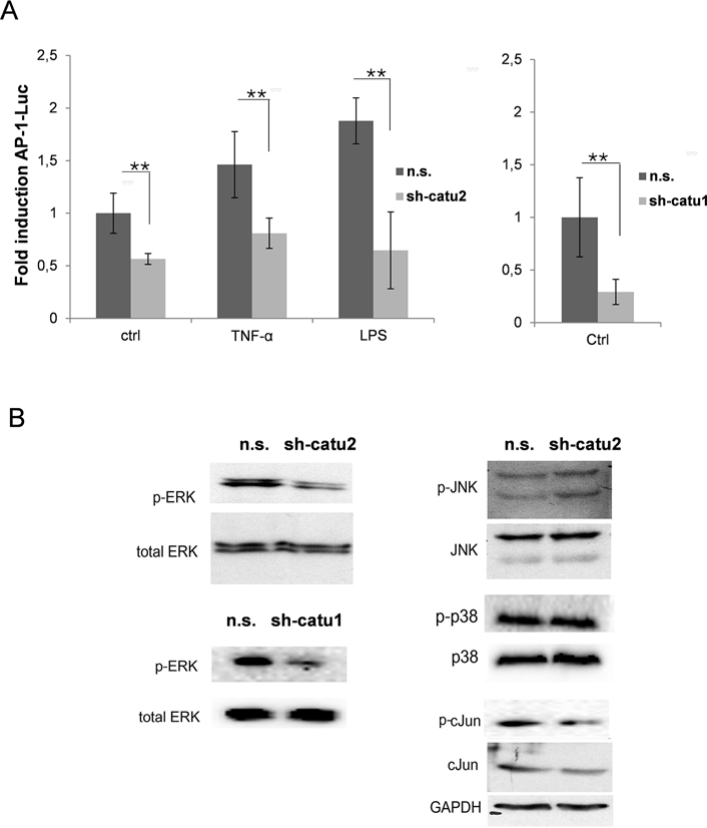
α-Catulin knockdown in melanoma cells reduces AP-1 and ERK activation. **(A)** An AP-1-luc reporter gene was transfected into non-stimulated stable infected Mel.7 cells (n.s., sh-catu1/2). The cells were non-stimulated (ctrl) or stimulated with TNF-α (10ng/ml) or LPS (5μg/ml). Luciferase levels were normalized for a co-transfected JRED control. (**B**) Mel.7 cells (as in **A**) were analyzed by Western blot with antibodies against phospho-ERK, total ERK, phospho-JNK, JNK, phospho-p38, p38, c-Jun and phospho-c-Jun. GAPDH was used as a loading control.

Together with NF-κB, AP-1 is known as an important transcription factor that regulates the expression of genes involved in inflammation, cell growth, survival and death [18, 28]. Having demonstrated that α-catulin plays a pivotal role in the activation of NF-ĸB, the next step was to determine the influence of α-catulin expression on AP-1 activity. Therefore, Mel.7 cells (n.s. and sh-catu1/2) were transfected with an AP-1 luciferase reporter gene and the cells were non-stimulated or stimulated with LPS or TNF-α. Down-regulation of α-catulin significantly decreased the basal AP-1-activity level and additionally reduced the AP-1 activity after stimulation with TNF-α or LPS in Mel.7 cells (Fig. 2A).

It is known that MAPKs are involved in the regulation of NF-κB and AP-1 activation [29, 30] and that they play a pivotal role in tumor cell growth, proliferation, apoptosis and survival [31]. Hence, we analyzed the effect of α-catulin knockdown on the activation of the MAPK family members ERK, JNK and p-38 using Western blot analysis. ERK phosphorylation was significantly reduced in Mel. 7 cells with α-catulin knockdown compared to control cells whereas JNK- and p-38 phosphorylation was not altered (Fig. 2B). The reduced phosphorylation and expression of c-Jun in α-catulin knockdown cells correlates with decreased AP-1 activity in the reporter assay (Fig. 2A).

Together these data indicate that the increased expression of α-catulin in malignant melanoma cells amplifies NFκB and AP-1 activity and the level of ERK phosphorylation.

### α-Catulin knockdown reduces ERK-, JNK- and c-Jun phosphorylation in cisplatin treated melanoma cells

It is known that cisplatin treatment induces several signaling pathways in melanoma cells, including NF-ĸB, MAPKs and apoptosis [6, 32]. Having demonstrated that α-catulin increased NF-ĸB, AP-1 activity and ERK phosphorylation in malignant melanoma cells (Figs. 1 and 2), we next examined how α-catulin influences NF-ĸB and MAPK signaling in cisplatin-treated melanoma cells.

**Fig 3:**
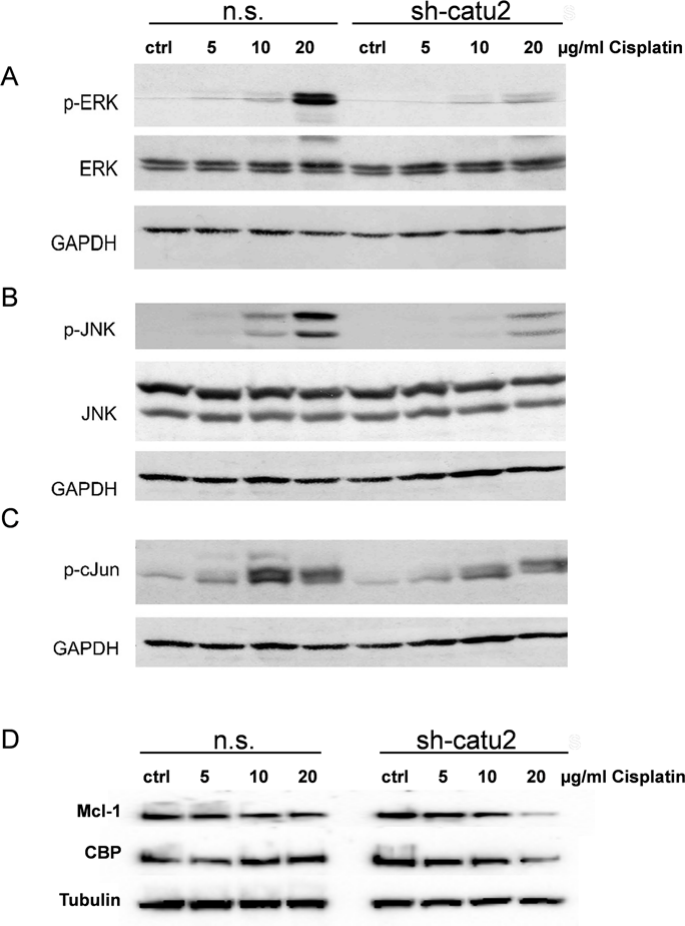
α-Catulin knockdown reduces phosphorylation of ERK, JNK and c-Jun in cisplatin treated melanoma cells. Stable infected Mel.7 cells (n.s., sh-catu2) were treated with 0, 5, 10 and 20 μg/ml cisplatin for 24h and analyzed by Western blot with antibodies against (**A**) p-ERK, total ERK, (**B**) p-JNK, total JNK, (**C**) p-c-Jun (**D**) Mcl-1 and CBP. GAPDH or Tubulin were used as loading control.

Mel.7 cells were treated (n.s. vs. sh-catu2) with different concentrations of cisplatin for 24 h and analysed for expression of p-ERK, p-JNK, p-cJun and the target genes Mcl-1 and CBP (CREB binding protein). Interestingly, the phosphorylation of the kinases ERK, JNK and c-Jun but also the protein level of Mcl-1 and CBP decreased dramatically in a dose dependent manner in α-catulin knockdown cells compared to control cells (Fig. 3A-D).

### α-Catulin knockdown sensitizes Melanoma cells to treatment with cisplatin

**Fig 4:**
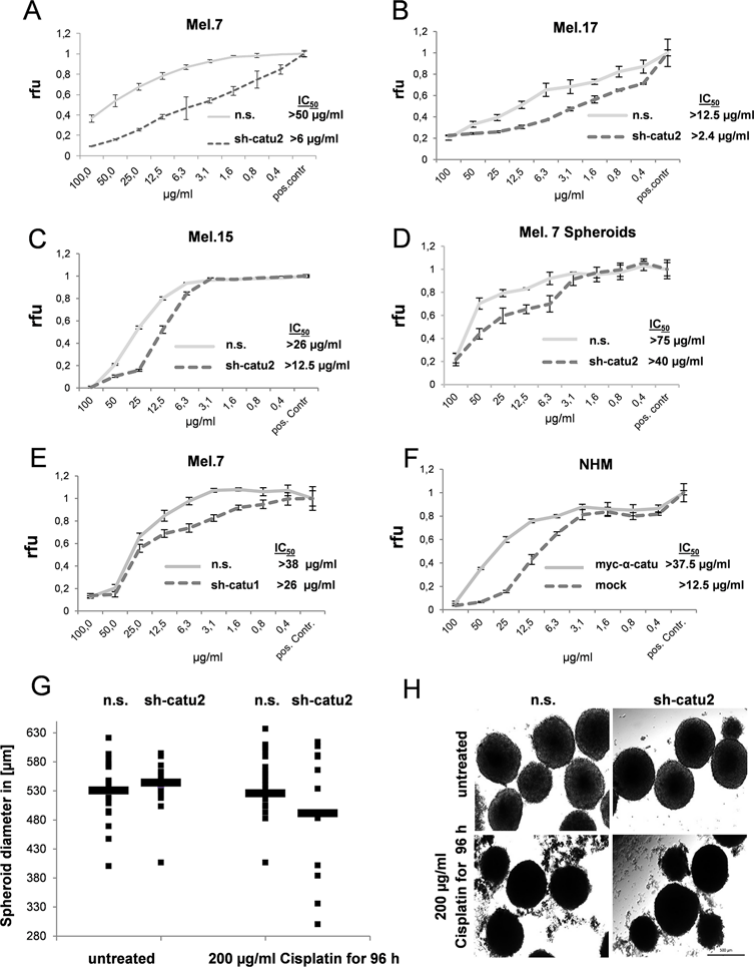
α-Catulin knockdown enhances susceptibility of melanoma cells to cisplatin. (**A-F**) Stable infected (n.s., sh-catu2) (**A**) Mel.7, (**B**) Mel.17, (**C**) Mel.15, (**D**) Mel.7 spheroids, (**E**) Mel.7 (n.s., sh-catu1) or (**F**) Melanocytes (mock, myc-α-catulin) were treated with different concentrations of cisplatin for 48h and cell survival normalized to untreated cells (pos. contr.). Viability was analyzed by CellTiter-Blue Assay. (**G**) Stable infected Mel.7 spheroids were treated with 200 μg/ml cisplatin for 96 hours and diameter of the spheroids determined before and after treatment and statistically evaluated. Observations (•) mean (-) n = 14, (**H**) Microscopic images from Mel.7 spheroids.

Since the previous results have shown that NFκB, AP-1 and ERK activity are increased in α-catulin expressing melanoma cells, we next sought to examine the effect of α-catulin knockdown on cell survival. Therefore, Mel.7, Mel.17 and Mel.15 cells (n.s. vs. sh-catu1/2) were treated with different concentrations of the chemotherapeutic drug cisplatin and cell survival was determined normalized to untreated control cells. In all tested cell lines α-catulin knockdown cells were 2–8.5-fold more sensitive to cisplatin treatment than cells expressing α-catulin. In particular, in Mel.7, Mel.15 and Mel.17 cells the IC-50 values were 50 μg/ml, 12.5 μg/ml and 26 μg/ml in control cells and 6 μg/ml, 2.4 μg/ml and 12.5 μg/ml in α-catulin knockdown (sh-catu2) cells after 48h treatment, respectively (Fig. 4A-C). These observations were additionally affirmed in Melanoma 7 cells transfected with sh-catu1 (Fig. 4E) and in primary melanocytes (NHMs) lentiviral transfected with myc-α-catulin and mock control (Fig. 4F). In order to confirm these observations in a more physiological assay we cultured Mel.7 cells as hanging drops to form spheroids and treated them for 48 hours with cisplatin. The determined IC-50 values were 75 μg/ml in control cells and 40 μg/ml in α-catulin silenced cells (Fig. 4D). To visualize this effect we treated the spheroids with an overdose of 200 μg/ml cisplatin for 96 hours and determined the size of the spheroids before and after treatment. Before treatment median size was slightly higher in α-catulin silenced cells compared to control cells. After treatment the size of α-catulin silenced spheroids was decreased dramatically, whereas non-silenced spheroids were only slightly smaller than before treatment (Fig. 4G, H).

To further elucidate the effect of α-catulin knockdown on other therapeutic agents, Mel.7 cells were treated with dacarbazine, paclitaxcel and the potent apoptosis inducer staurosporine for 48h. α-Catulin knockdown reduced cell survival also significantly for these chemotherapeutic drugs (S2 A–C Fig.).

### α-Catulin knockdown decreases cell proliferation in cisplatin-treated melanoma cells

**Fig 5:**
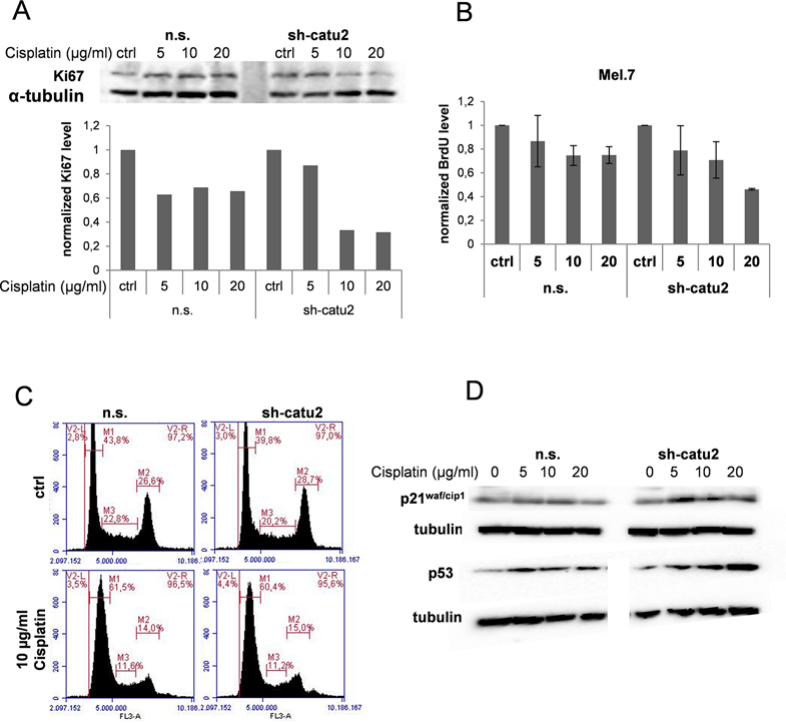
Cell proliferation is reduced in a dose- and time dependent manner in cisplatin treated melanoma cells when α-catulin is knocked down. (**A**) Stable infected Mel.7 cells (n.s., sh-catu2) were treated with 0, 5, 10 and 20 μg/ml cisplatin for 24 h and analyzed by Western blot with antibody against Ki67. α-Tubulin was used as a loading control and quantification was performed with BioRad software. (**B**) Mel.7 cells (n.s., sh-catu2) were treated with 20, 10, 5, or 0 μg/ml cisplatin for 48 h and BrdU assay was performed. Therefore, cells were stained with BrdU solution and antibodies against BrdU and HRP conjugated secondary antibody was detected at 450 nm using a multiplate reader. (**C**) Mel.7 cells (n.s., sh-catu2) were treated with 0 or 10 μg/ml cisplatin for 18 hours, fixed, stained with propidium-iodide solution and analysed for cell cycle distribution using flow cytometry. (**D**) Cells as in (**C**) were analysed using western blot with antibodies against p21^cip/waf^ and p53.

AP-1, NFκB and ERK are also important regulators of cell proliferation [15, 18, 31]. To elucidate the influence of α-catulin and cisplatin treatment on cell proliferation we treated Mel. 7 cells with different concentrations of cisplatin and analyzed them for expression of the proliferation marker Ki-67 using Western blot. Ki-67 expression was significantly reduced in α-catulin knockdown cells treated with 10 or 20 μg/ml cisplatin compared to control cells (Fig. 5A). To further investigate this observation we treated the cells with 5, 10 or 20 μg/ml cisplatin or left them untreated (ctrl) and BrdU Assay was performed as described in Material and Methods. BrdU incorporation was slightly reduced in cisplatin treated Mel. 7 cells compared to the untreated cells (Fig. 5B). Additionally, the impaired proliferation in α-catulin knockdown cells treated with 20 μg/ml cisplatin compared to non-silenced control cells was confirmed suggesting that knockdown of α-catulin reduces cell proliferation in cisplatin-treated melanoma cells. To further confirm these observations we analysed the cell cycle distribution in Melanoma 7 cells treated with 0 or 10 μg/ml cisplatin for 48 hours (Fig. 5C). Compared to the untreated controls, the relative amount of cells in G1 was enhanced, whereas the percentage of cells in G2 and S-phase was significantly reduced in both n.s. and sh-catu2 cells after cisplatin treatment. Further, we analysed the influence of α-catulin knockdown on the cell cycle inhibitors p21^waf/cip1^ and p53 in cisplatin treated Mel. 7 cells. Fig. 5D clearly demonstrate a dose dependent expression of p21^waf/cip1^ and p53 in sh-catu2 melanoma cells after cisplatin treatment.

### α-Catulin knockdown enhances apoptosis in cisplatin-treated melanoma cells

**Fig 6:**
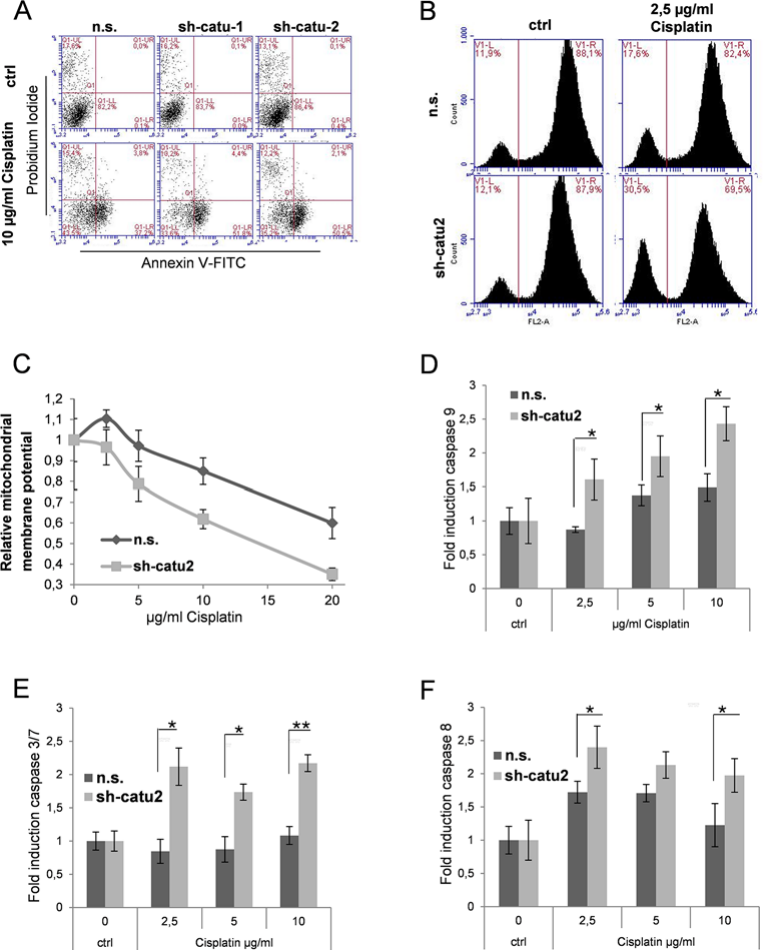
α-Catulin knockdown enhances apoptosis in cisplatin-treated melanoma cells. (**A**) Stable infected Mel.7 cells (n.s., sh-catu1, sh-catu2) were treated with 0 and 10 μg/ml cisplatin for 48 h and stained with Annexin V and PI and analysed using flow cytometry (**B**) Stable infected Mel.7 cells (n.s., sh-catu2) were treated with 0 and 2.5μg/ml cisplatin for 6 h. For cytochrome c release assay, cells were treated with permeabilization buffer, fixed with formaldehyde, stained with antibody against cytochrome c and analyzed by Flow Cytometry. (**C**) Mel.7 cells (n.s., sh-catu2) were seeded in a 96 well plate and treated with 0, 2.5, 5, 10 or 20 μg/ml cisplatin for 6 h and mitochondrial membrane potential was determined using JC-1 assay (**D**) Mel.7 cells (n.s., sh-catu2) were treated with 0, 2.5, 5 or 10 μg/ml cisplatin for 6 h and analyzed for caspase 9 activity using caspase glo luminescence assay. (**E**) Cells as in (**D**) were analyzed for caspase 3/7 and (**F**) caspase 8.

Measuring cell metabolism, we clearly demonstrated that α-catulin knockdown melanoma cells are more susceptible to cisplatin treatment than control cells (Fig. 4). To further investigate the mechanism of cell death, we evaluated the AnnexinV- and propidium iodide (PI)-positive cells. As depicted in Fig. 6A, apoptosis (Annexin V staining) was significantly enhanced in α-catulin knockdown cells (sh-catu1: 56.2% and sh-catu2: 52.6%) compared to control cells (n.s.: 41%) after cisplatin treatment for 48 h. No striking differences were observed in necrotic cell (PI-positiv).

It has been reported that levels of cytochrome c in the cytoplasm were enhanced in cisplatin treated cells [33]. Cytochrome c release from mitochondria is an early event when cells undergo apoptosis [34]. To investigate the contribution of α-catulin on cell death after treatment with cisplatin we performed a cytochrome c release assay and compared α-catulin knockdown melanoma cells with control cells. Mel. 7 cells were untreated or treated for 6 hours with 2.5 μg/ml cisplatin. Then the cells were fixed, permeabilized and stained with a specific antibody against cytochrome c as described in Material and Methods, followed by flow cytometry analysis. Release of cytochrome c from mitochondria in the course of apoptosis results in a wash-out from the permeabilized cells and reduction of cytochrome c-related fluorescence. In the absence of cisplatin, the percentage of apoptotic cells was almost equal for control and α-Catulin knockdown cells with 11.9% and 12.1%, respectively. However, α-Catulin knockdown significantly increased the percentage of apoptotic cells after cisplatin treatment with 30.5% compared to control cells (n.s.) with 17.6% apoptotic cells (Fig. 6B).

Along with cytochrome c release the mitochondrial membrane is depolarized in cisplatin induced apoptosis [35, 36]. To investigate the effect of α-catulin knockdown on mitochondrial membrane potential in cisplatin treated melanoma cells, we used the fluorescent JC-1 dye. The relative mitochondrial potential depolarized in a dose-dependent manner in both α-catulin knockdown and controls, however, was significantly lower in α-catulin knockdown cells (Fig. 6C).

In the cytoplasm cytochrome c binds to APAF-1 and then activates caspase-9 and further caspase-3/7 [37]. To analyse whether these steps of the intrinsic apoptosis cascade are also influenced by α-catulin we treated the cells with different concentrations of cisplatin and detected caspase-9 and-3/7 using a caspase luminescence assay. Fig. 6 demonstrates that caspase-9 and caspase-3/7 activities were significantly enhanced in α-catulin-knockdown cells treated with 2.5, 5 or 10 μg/ml cisplatin after 6 hours (Fig. 6D and E). As described previously, cisplatin can also induce apoptosis via extrinsic pathways which means that cisplatin-resistance can only be achieved when both the intrinsic and the extrinsic apoptotic pathway are inhibited [38]. We therefore performed a caspase-8 luminescence assay and found that α-catulin-knockdown also increased caspase-8 activity (Fig. 6F).

Taken together, these results indicate that gene suppression of α-catulin in malignant melanoma cells enhances cisplatin-induced apoptosis in both the intrinsic and the extrinsic pathways. These findings suggest that the high level of α-catulin in melanoma cells contributes to drug resistance against cisplatin treatment.

## Discussion

Cisplatin was described as one of the most potent antitumor agents with clinical activity against many different cancers [6–8]. The covalent binding of cisplatin to chromosomal DNA leads to the formation of DNA adducts and activates several signal transduction pathways contributing to apoptosis [39]. Resistance of tumor cells to chemotherapeutic agents is a limiting factor of chemotherapy and therefore a better understanding of the mechanisms of chemoresistance is of great interest [6].

In this study, we showed that high expression of α-catulin played a critical role in resistance of melanoma cells to cisplatin. First, we demonstrated that melanoma cells exhibit a significantly higher expression of α-catulin and a concomitant elevated activity of the transcription factor NF-ĸB (Fig. 1). In opposition to malignant melanoma cells, normal human melanocytes (NHM) showed no or only low NF-ĸB activity and a low α-catulin expression level (Fig. 1) [24]. Transfection of α-catulin in melanocytes augmented NF-ĸB activity in a dose dependent manner, whereas α-catulin knockdown reduced it. Significant NF-ĸB activity was found in α-catulin expressing cells after stimulation with TNF-α, LPS, HGF or Serum. Co-transfection of α-catulin with IKKβ demonstrated a significant NF-ĸB activation in α-catulin expressing cells whereas co-transfection of α-catulin with p65 had only a very low effect, confirming that α-catulin operates upstream of p65 in the NF-ĸB signaling pathway [23]. Furthermore, the result proved that α-catulin activate NF-ĸB by repressing E-cadherin, a fundamental event in EMT. There is ample evidence that α-catulin plays an important role in tumorigenesis [23, 24, 40, 41], and that activation of NF-ĸB pathway is the major signal for the induction of EMT, a developmental mechanism that is characterized by loss of cell-cell adhesion and polarity followed by a disruption of cytoskeletal organization toward a more mesenchymal phenotype [42–45]. EMT has been closely linked to progression and invasion of different tumors and has been described to be regulated by MAPK-ERK signaling pathways by activation of ZEB1/2 via Fra1, which is an important member of the AP-1 transcription factor complex [46]. Additionally, activation of ZEB1/2 has been connected to NF-ĸB signaling in breast cancer cells [47], suggesting an involvement of the MAPK, NF-ĸB and AP-1 pathways in tumorigenesis through activation of EMT.

Furthermore, MAPK, NF-ĸB and AP-1 are known to play an important role in cisplatin-mediated apoptosis [48–50]. Therefore, we sought to examine the effect of α-catulin knockdown on the activation of AP-1 and the phosphorylation of ERK, JNK and p38. Fig. 2 depicts that α-catulin knockdown reduced AP-1 activation and ERK phosphorylation. However, there are controversial reports about the contribution of MAPK to apoptosis or cell survival depending on the cell type and extent of DNA damage. Dent and Grant (2001) reported that activation of ERK and JNK MAPK cascades by cisplatin counteract apoptosis [48]. Agreeing with this, Persons et al. (1999) showed that ERK inhibition increased the cisplatin-sensitivity of ovarian cancer by accumulating p53 [51]. In contrast, Wang et al. (2000) showed that ERK activation is essential for cisplatin-mediated apoptosis in HeLa and lung carcinoma cells [21]. Therefore, we were interested how α-catulin influences different signaling pathways in cisplatin-treated melanoma cells and whether α-catulin knockdown leads to increased sensitivity to chemotherapeutics. Hence, the cisplatin treated cells were analyzed for NF-ĸB, MAPK and AP-1 signaling. In cisplatin treated melanoma cells, α-catulin knockdown reduced NF-ĸB activity, ERK-, JNK- and c-Jun phosphorylation and the protein level of the target genes Mcl-1 and CBP (Fig. 3). Consistent with our findings Mirmohammadsadegh et al. (2007) reported that increased phosphorylation of ERK1/2 in melanoma cells promoted tumor progression and partially prevented cisplatin-mediated apoptosis [32]. As a consequence, we treated the melanoma cells with the chemotherapeutic drugs cisplatin, dacarbazine and paclitaxcel to elucidate the effect of α-catulin knockdown along with reduced AP-1 activatioin and ERK and JNK phosphorylation in melanoma cells. α-Catulin knockdown enhanced the susceptibility of melanoma cells to the chemotherapeutic drugs cisplatin, dacarbazine and paclitaxcel (Fig. 4,S2 Fig.). This result suggests that NF-ĸB and AP-1 activation and ERK phosphorylation leads to reduced cell death in melanoma cells after treatment with chemotherapeutic agents. Accordingly, Mandic et al. (2001) reported that ERK acted as a pro-survival protein in melanoma cells [52]. Additionally, we treated the cells with staurosporine, a potent inducer of apoptosis, which also showed increased sensitivity of α-catulin knockdown cells (S2 Fig.).

The increased expression of NF-ĸB, p-ERK and AP-1 due to cisplatin treatment raised the question, whether cisplatin treatment leads to enhanced proliferation in melanoma cells. Therefore, we analyzed Ki67 expression and found that cell proliferation was not altered due to cisplatin treatment in melanoma cells expressing α-catulin. However, Ki67 expression was significantly reduced in α-catulin knockdown cells treated with higher concentrations of cisplatin (Fig. 5). Same results could be confirmed with the BrdU Assay suggesting that α-catulin knockdown reduces cell proliferation in cisplatin-treated melanoma cells. Furthermore, the expression of the cell cycle inhibitors p21^waf/cip1^ and p53 demonstrated a dose dependent increase in α-catulin knockdown cells after cisplatin treatment.

Next, we investigated the role of α-catulin in cisplatin induced apoptosis. α-Catulin knockdown significantly enhanced apoptosis after cisplatin treatment (Fig. 6). Cytochrome c release from mitochondria was described as a major step in the induction of apoptosis mediated by cisplatin [33]. Therefore, we clearly demonstrated that cytochrome c release was increased in α-catulin knockdown cells after cisplatin treatment and that this effect was accompanied by mitochondrial membrane depolarization, caspase-9, -8 and-3/7 activation (Fig. 6).

## Conclusions

In summary we show in this study, that the high level of α-catulin in melanoma is responsible for NF-κB, AP-1 activation and ERK phosphorylation and contributes to a reduction in cisplatin-mediated apoptosis. α-catulin knockdown reduced cisplatin-mediated cell proliferation and enhanced apoptosis in melanoma cells compared to the control cells. Together with our previous findings, these results demonstrate that α-catulin plays an important role in tumor progression, metastasis and chemoresistance suggesting α-catulin as a novel promising target for melanoma therapy.

## Supporting Information

S1 Fig Transfection efficiency into primary melanocytes using Lipofectamin 2000. Primary melanocytes were mock (2 μg) transfected or co-transfected with α-catulin (1 μg), IKK-β (0.25 μg) and p65 (0.25 μg) or non-transfected. Transfection efficiency was analysed by western blot using antibodies against IKK-ß, α-catulin, p65 and tubulin (loading control).
(TIF)

S2 Fig α-Catulin knockdown enhances susceptibility of melanoma cells to chemotherapeutic drugs. Stable infected Mel.7 (n.s., sh-catu2) cells were treated with different concentrations of (**A**) Dacarbazine, (**B**) Paclitaxel or (**C**) Staurosporine for 48h and cell survival normalized to untreated cells (pos. contr.). Viability was analyzed by CellTiter-Blue Assay.
(TIF)
