# Supplementary figures and images for: Alpha-Catulin Contributes to Drug-Resistance of Melanoma by Activating NF-κB and AP-1
Source: PLoS One. 2015 Mar 20;10(3):e0119402. doi: 10.1371/journal.pone.0119402 (PMC4368766; doi:10.1371/journal.pone.0119402)

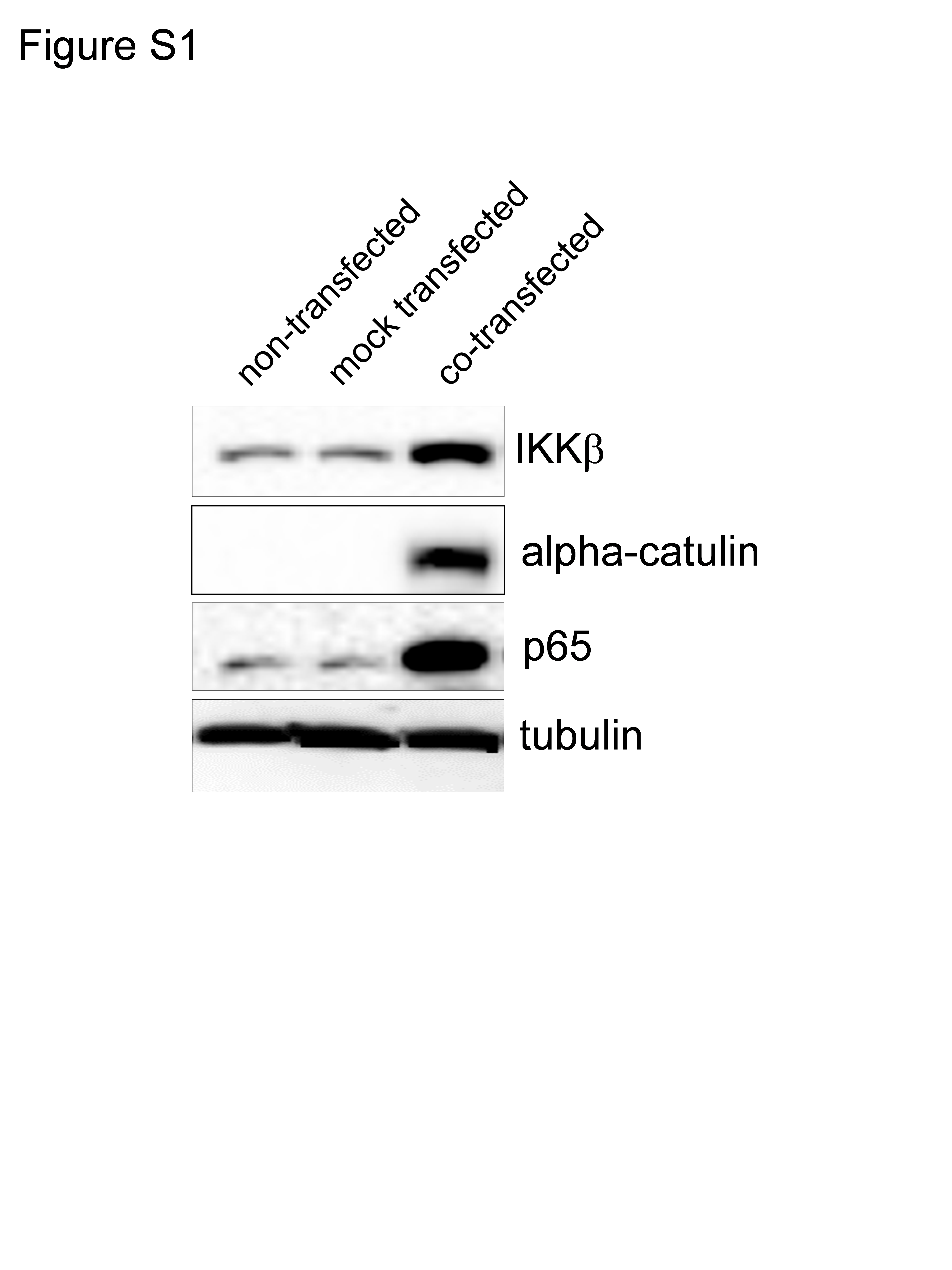

Supplement: S1 Fig — Primary melanocytes were mock (2 μg) transfected or co-transfected with α-catulin (1 μg), IKK-β (0.25 μg) and p65 (0.25 μg) or non-transfected. Transfection efficiency was analysed by western blot using antibodies against IKK-ß, α-catulin, p65 and tubulin (loading control). (TIF) [file pone.0119402.s001.tif]

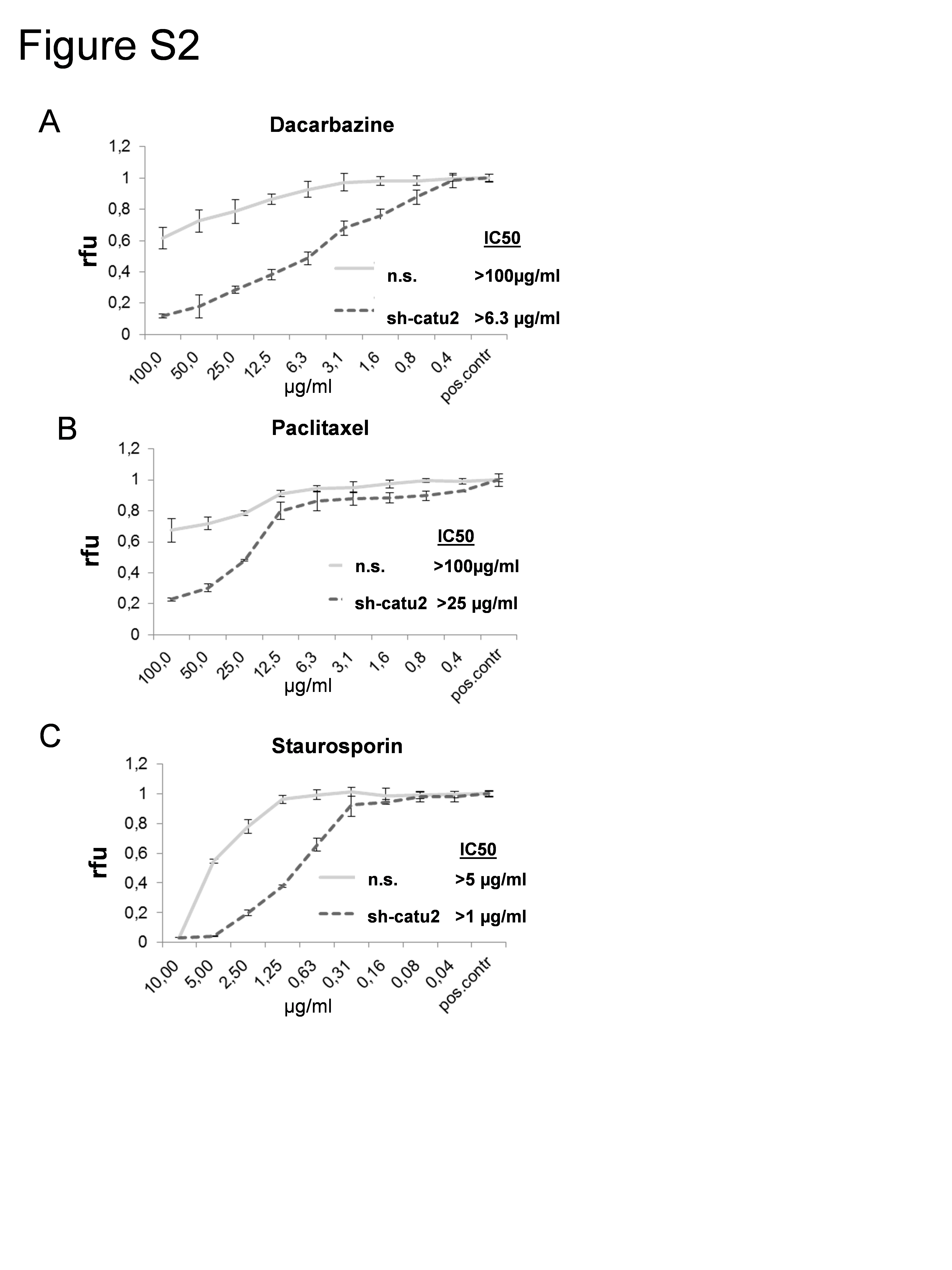

Supplement: S2 Fig — Stable infected Mel.7 (n.s., sh-catu2) cells were treated with different concentrations of (A) Dacarbazine, (B) Paclitaxel or (C) Staurosporine for 48h and cell survival normalized to untreated cells (pos. contr.). Viability was analyzed by CellTiter-Blue Assay. (TIF) [file pone.0119402.s002.tif]
